# Convolutional neural network-based classification system design with compressed wireless sensor network images

**DOI:** 10.1371/journal.pone.0196251

**Published:** 2018-05-08

**Authors:** Jungmo Ahn, JaeYeon Park, Donghwan Park, Jeongyeup Paek, JeongGil Ko

**Affiliations:** 1 Department of Computer Engineering, Ajou University, Suwon, Republic of Korea; 2 Electronics and Telecommunications Research Institute, Daejeon, Republic of Korea; 3 School of Computer Science & Engineering, Chung-Ang University, Seoul, Republic of Korea; Chongqing University, CHINA

## Abstract

With the introduction of various advanced deep learning algorithms, initiatives for image classification systems have transitioned over from traditional machine learning algorithms (e.g., SVM) to Convolutional Neural Networks (CNNs) using deep learning software tools. A prerequisite in applying CNN to real world applications is a system that collects meaningful and useful data. For such purposes, Wireless Image Sensor Networks (WISNs), that are capable of monitoring natural environment phenomena using tiny and low-power cameras on resource-limited embedded devices, can be considered as an effective means of data collection. However, with limited battery resources, sending high-resolution raw images to the backend server is a burdensome task that has direct impact on network lifetime. To address this problem, we propose an energy-efficient pre- and post- processing mechanism using image resizing and color quantization that can significantly reduce the amount of data transferred while maintaining the classification accuracy in the CNN at the backend server. We show that, if well designed, an image in its highly compressed form can be well-classified with a CNN model trained in advance using adequately compressed data. Our evaluation using a real image dataset shows that an embedded device can reduce the amount of transmitted data by ∼71% while maintaining a classification accuracy of ∼98%. Under the same conditions, this process naturally reduces energy consumption by ∼71% compared to a WISN that sends the original uncompressed images.

## 1 Introduction

For many years, various wireless sensor networks (WSNs) have been deployed to digitalize and understand many physical aspects in the real-world. Early generation of WSNs focused mostly on data collection where the goal of these deployments were simply to gather data, report these information to the data managing personnel, and allow for offline analysis on the data [1–3]. Following generation of WSNs started to involve some form of in-network processing. The purpose of in-network processing was to reduce unnecessary transmission of redundant data and collect improved-quality data by pre-processing the data closer to the data source. Furthermore, in some cases, this processing led to multiple levels of actuation in the target environment [4]. Nevertheless, energy efficiency of the devices has been the key concern for WSNs, and researchers have proposed many schemes to minimize the energy usage on the battery operated, resource limited sensing platforms (e.g., motes [5, 6]). While many WSN deployments began with simple sensing modalities such as temperature and humidity sensors, the improvements in processing power allowed the introduction of more advanced sensing modalities such as cameras, introducing wireless image sensor networks (WISNs).

**Fig 1:**
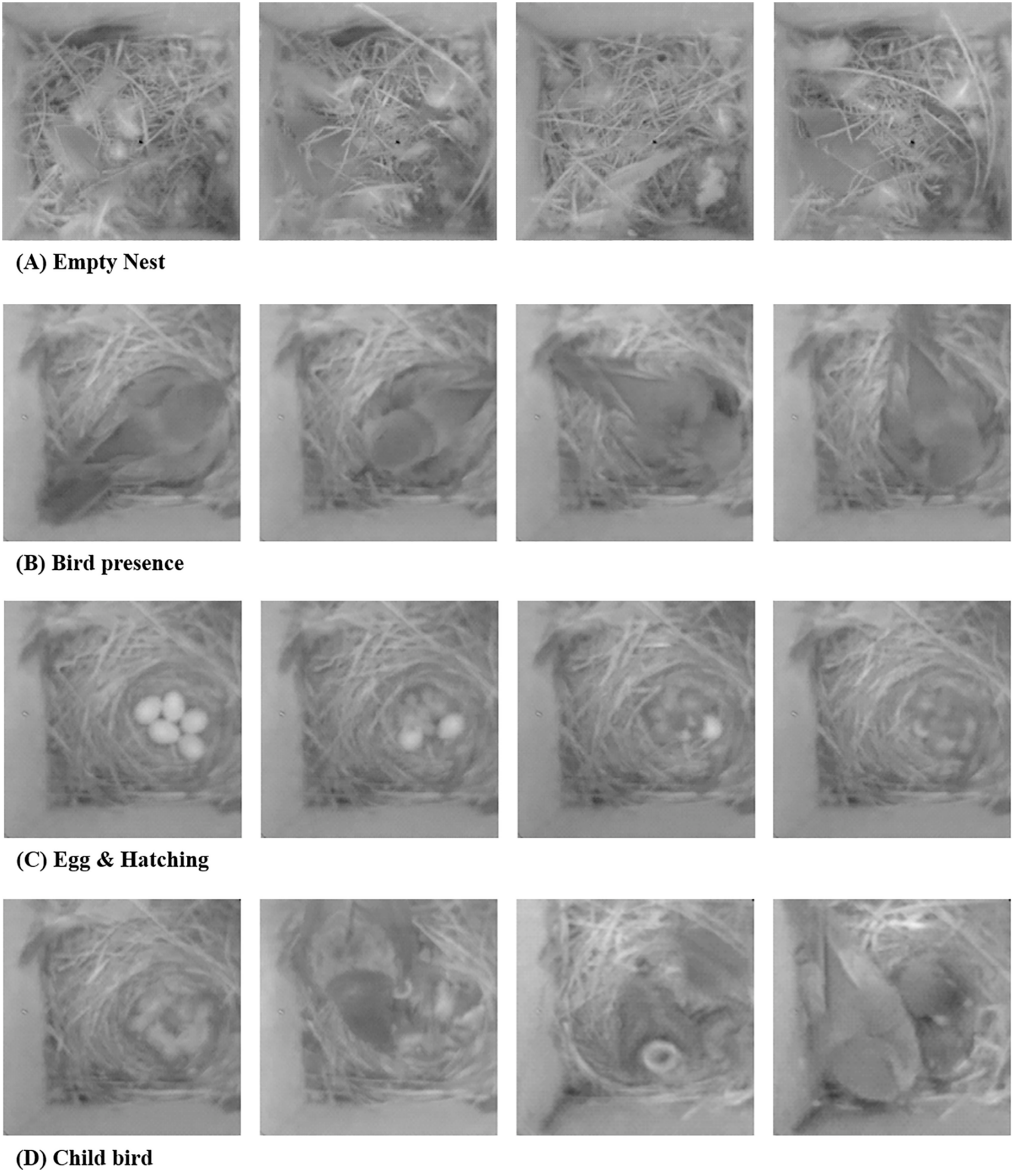
Sample bird nest images [7] from a WISN deployment in [8].

WISNs are typically implemented on low-power, low-cost embedded platforms such as Cyclops [9], FireFly Mosaic [10] and WiSN [11]. These networks are used for applications such as surveillance systems [12], environmental monitoring systems [13, 14] and animal habitat monitoring applications [8, 15, 16]. In addition to sensor networks, recent advances in machine learning and deep learning algorithms are providing a software platform for domain scientists to better understand the data collected from various environments [17–19]. Unlike traditional image classification algorithms (e.g. Support vector machine (SVM), Multi-layer perceptron (MLP), K-Nearest Neighbors (K-NN)), deep neural networks’ strength is in the fact that it allows for a full autonomous operation since these schemes extract data-related features with minimal human input or prior knowledge of the data. Nevertheless, learning algorithms are still very complex and are challenging to apply in WSNs directly due to their computational overhead and energy constraints. Instead, these data analytics tools can function on the backend server if the sensor nodes can collect enough data for the model training process. However, to train these machine learning models, large amount of data points are required. For WSNs that collect small-sized sensing information, this data collection process may be less of an overhead, while sensor networks that focus on multimedia contents, such as images, may need to use too much of its resources just to collect effective training data points. Given the limited resources on the embedded sensing platforms, WISNs may not be able to produce high-resolution images, and thus the images may be even difficult for humans to distinguish and understand in some cases (e.g. Fig 1).

In this paper, we present an autonomous environmental monitoring system composed of a WISN and a CNN-based image classifier [20]. WISN is responsible for collecting image data with low-power camera sensor under battery-limited environment, and the CNN-based image classifier classifies the content of the captured images from sensor nodes using a classification model based on deep neural network. To showcase the effectiveness of our proposed system, we use images collected from a real-world field deployment for bird nest monitoring at James Reserve [8]. This dataset contains 102,173 images of the interior of bird nests [7]. The goal of our proposed system is to understand the context of each bird nest autonomously so that accurate information is reported to the domain scientists.

We first start this work by trying to understand the overhead of gathering the full data set of original raw images for usage in CNNs. Given that the images are *not small* in size, the overhead of exchanging image data, potentially over multiple hops, can negatively impact the system lifetime. To minimize the energy usage and transmission overhead of exchanging raw images with the data analytics server, this work introduces a set of image pre-processing techniques which is essentially a lossy image compression algorithm that combines color quantization and image resizing techniques. We then show that our design of the compression scheme does not negatively impact the classification accuracy of the CNN model.

The focus of our system design is to make sure that we have a compressed image that can lessen the transmission overhead of image-containing packet transmissions, while keeping the complexity of the image pre-processing algorithms to a reasonable level. Despite dropping the quality of the images collected from the WISNs, we show that intelligently compressing these images can maintain the context detection accuracy of a CNN at ∼98% while reducing network transmission overhead by ∼71%.

The contributions of this work is as follows.

- We identify the high energy consumption problem in the image collection-classification process on wireless image sensor network and suggest a system scenario for energy efficient operation.
- We design a low computational overhead lossy image compression scheme for embedded devices in wireless image sensor network to reduce battery consumption of WISN nodes significantly, while maintaining the classification accuracy of CNN at the backend server. Our design combines color quantization and resizing techniques appropriately, for training and testing data, to achieve this goal.
- We apply our approach to image dataset from real world deployments of wireless image sensor network and show that our approach achieves high accuracy along with high compression ratios. We also identify that convolutional neural networks work robustly on top of lossy image compression algorithm.

The remainder of this paper is structured as follows. In Section 2, we introduce our target scenario and system design to identify how our proposed system would be used. In Sections 3 and 4, we introduce our proposed lossy image compression scheme and show its impact from the perspective of distortion and battery consumption, respectively. Section 5 presents the CNN model using the lossy-compressed images and describe how our model is built. Here, we also evaluate the classification accuracy of our system. Finally, we discuss related work in Section 6 and conclude this paper in Section 7.

## 2 Scenario and system design

Many wireless sensor networks require a classification of the incoming data as a way for domain scientists to better understand the collected data, or with the purpose of performing different types of actuation in the environment. For wireless image sensor networks where embedded devices deployed at many geological points gather a large amount of image data, providing a contextual summary or classification result can help researchers easily understand what is occurring in the environment. However, nodes used in wireless sensing systems are typically resource-scarce. The widely used TelosB mote platform has a 16bit MCU with 8 MHz clock, 10 KB of RAM and 48 KB of flash [5, 21]. Merely being enough to capture and store a single image, complex operations cannot take place on these devices themselves. For this reason, sensor nodes equipped with a small-sized low-resolution camera will typically report their sensor readings to the backend server, while the server performs some form of image classification and/or acts as a data repository.

On the other hand, recent advances in various deep learning algorithms and tools made it seem plausible to approach such a system using deep learning. To train a neural network at the backend server, it is important and widely thought of that a set of high quality images are required, potentially in large quantities. However, for resource limited wireless sensor networks, providing such image data set itself can be a burden. Note that sensor networks deployed in the environment typically target for uninterrupted system lifetimes of multiple months to years. Sending raw, high-resolution image data using wireless radios can quickly drain the devices’ batteries and decrease the system lifetime significantly.

To this end, this work focuses on maximizing the effectiveness of a wireless image sensor network that uses deep learning algorithms for image classification at the backend data collection server. We propose to send compressed images to the backend server using *lossy* compression which achieves high compression ratios, but keep the loss of quality minimal to the level which the backend deep learning classifier can make proper decisions. This is done using two core techniques, namely color quantization and image scaling, and applying them adequately and differently for training and test data.

**Fig 2:**
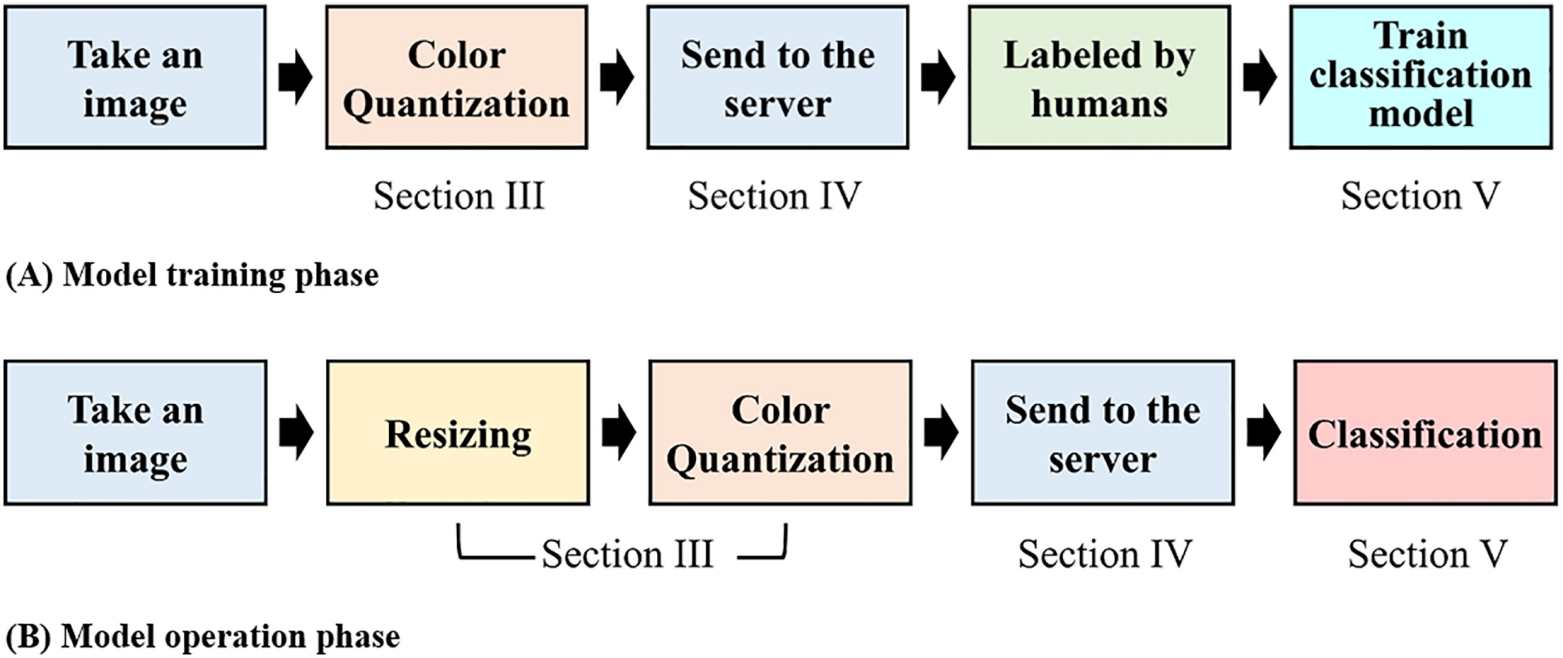
System pipeline for (a) model training phase, and (b) model operation phase.

Fig 2 illustrates the flow of our system as two different pipeline phases. First, as the Fig 2(a) shows, a level of human interaction is required to provide the ground truth for training the CNN model. Note that, as we will detail in Section 3, nodes perform color quantization on the raw images prior to sending the data. With this “compressed” data, domain scientists can label the images on the backend server prior to training the CNN model. This is a human-involved process that cannot be avoided in training machine learning models. We agree that it may be more accurate for domain scientists to label uncompressed original images rather than the compressed images. Nevertheless, lower quality images can be “good enough” for the labeling purpose, and they can also allow the system to be more efficient (e.g., use less energy/bandwidth resources). We will present the visual quality of the compressed images in Section 3, suggesting that compression will minimally affect the ground-truth labeling process. On the other hand, when sending data during normal operations (e.g., the CNN model is pre-trained and classification is in progress), an additional step of image resizing takes place at the sensor node to further reduce the image size for energy efficient transmissions (c.f., Fig 2(b)).

**Fig 3:**
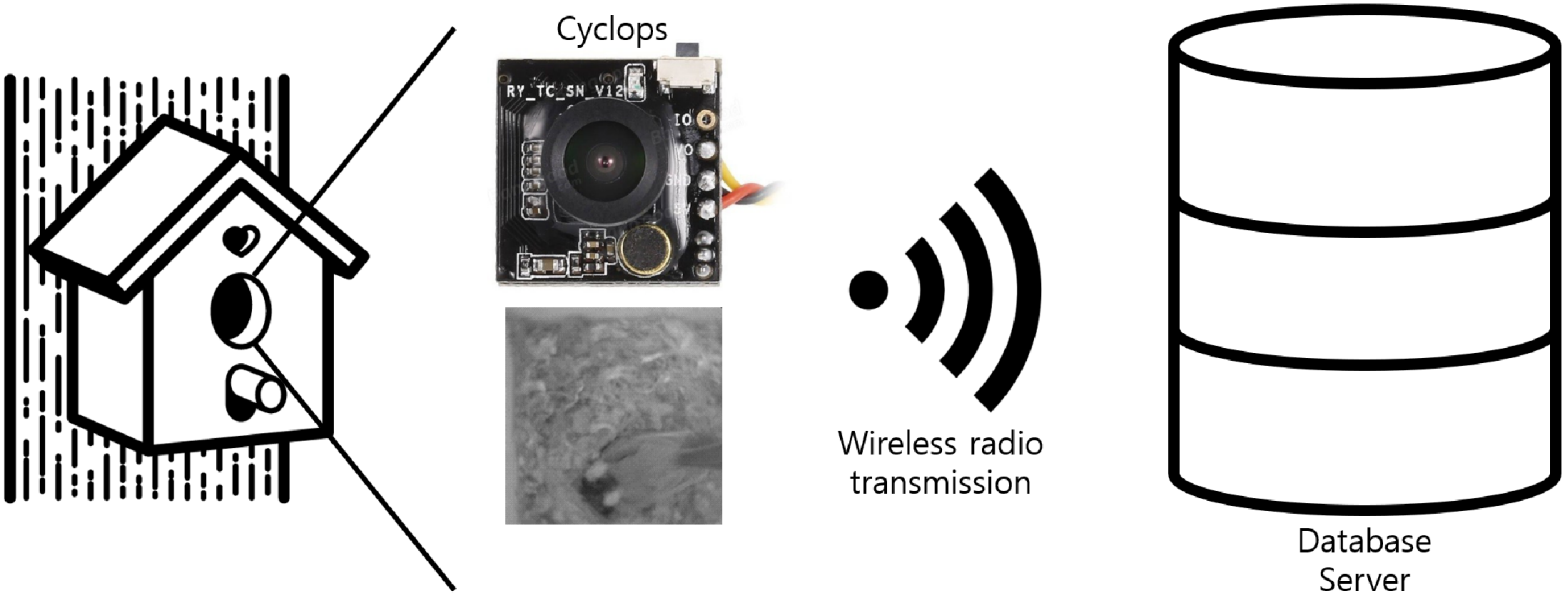
System architecture for bird nest monitoring [7].

As a application example to better motivate and explain our system, we choose a bird nest monitoring system as the target application [8, 15]. Using this scenario and the images as an example, we will discuss how the proposed image classification pipeline for WISNs can provide an energy efficient method for implementing various image-based sensing & classification systems. Specifically, we use the 102,273 images collected from the work by Paek et al. [7, 8], in which the authors developed the Cyclops platform to monitor bird nests at the James San Jacinto Mountain Reserve. The work was done with a tight collaboration with avian biologists who tried to understand the activity of birds in their nests. The project installed a pair of Cyclops and motes on 19 bird nests and captured 200 × 200 pixel 8 bits grayscale images of the nest periodically every ∼15 minutes. As illustrated in Fig 3, the images were transmitted to a backend server, possibly over multihop, and were stored for a system deployment duration of 4 months. From these collected images, our goal is to classify them as {“Empty Nest”, “Bird Presence”, “Egg & Hatching” and “Child bird”} as shown in Fig 1.

In the work by Ko et al. [15], the authors perform bird nest context classification using traditional machine learning algorithms, such as feature key point (SIFT) based support vector machine (SVM) and Hidden Markov Models. While this approach showed good accuracy (e.g., 82% and 84% for bird presence/absence and egg counting, respectively), it required raw (best possible quality) images to be sent to the server at all times. Our approach tries to lessen the transmission overhead of battery limited sensor nodes by performing a level of in-network processing to compress the image size prior to transmissions. While this approach adds additional complexity to the sensor nodes, as we show later in Section 3, since radio transmissions are a dominant energy user in typical wireless sensor motes, this leads to a meaningful amount of energy usage reduction, leading to longer system lifetimes.

Based on our observations and lessons learned from previous work, the design goals of our system are as follows.

- To reduce energy consumption on wireless sensor nodes, each node should reduce the amount of data being sent to the backend server. This suggests that some form on in-network data compression is needed in the system.
- Compressed images from the nodes should be of sufficient quality to be annotated (or labeled) by domain experts. This means that excessive lossy compression is not an option for our system. Thus, we compare the SSIM of the compressed images with the original image.
- An autonomous monitoring system should be able to make classification decisions using the compressed images (only). Therefore, there is a need for a comprehensive study on how much we can compress the images without loss of classification accuracy.
- When nodes capture images with their camera module, the images are often broken due to the limited resources on the nodes. Such outlier images confuse the classification model; thus, there should be a mechanism to filter out corrupted images.

The sections that follow provide details on how images are processed on the wireless sensor nodes to perform size compression for the sake of minimizing the packet transmission overhead.

## 3 Decreasing resolution

An effective way to reduce the size of data transmitted over the radio is to compress the image itself. While the images taken from the deployment of our interest is only 200 × 200 pixels in resolution, the size of a single image (40 KB) is still relatively larger than what these platforms were designed for. Therefore, reducing the pixel count further is a direct way to significantly reduce the energy usage of the resource-limited nodes. Even if we had more advanced platforms, same approach would apply since we would then want relatively higher resolution images. Additionally, given that each pixel is represented using 256 grayscale, performing color quantization to reduce the number of bits that represent a single pixel can also help reduce the image size. Again, same approach applies even if we have 256 × 256 × 256 RGB colors. This section focuses on providing details on these two mechanisms that are used to reduce the image size.

Despite reducing the size, a design goal of our scheme is to maintain high image similarity with the original image. This is important for both the ground truth labeling and model training phase, and also for the operational phase as well. For the sake of comparing the image quality of the compressed image with the original image, we calculate and compare the structural similarity (SSIM) index of the two images. SSIM is a widely-used similarity metric between two images [22]. Generally, the SSIM index is used to identify similarity between an original image and a distorted image or the reconstruction of a lossy compressed image. This metric is known to be more effective than mean square error (MSE) or peak signal-to-noise ratio (PSNR). SSIM is a normalized metric on a range from zero to one so that it is easy to recognize how compressed images are distorted (i.e., higher SSIM means lower distortion). Another reason for using the SSIM index is that we train and classify with the *compressed* images on the CNN at the backend sever. Note that the first layer of the CNN architecture is a convolutional layer which is designed for identifying low-level features such as colors, edges and curves [23]. Therefore, the SSIM index, which is a metric that compares the similarity of *structural* characteristics, is more suitable in identifying the quality degradation, if any, compared to other image quality representation metrics [24].

### 3.1 Color quantization

Color quantization is a process that tries to reduce the number of colors that represent a single pixel. Specifically, this process reduces the number of colors on a “palette” and re-draws the image using the limited number of colors on the palette. This palette represents a finite set of colors that a single pixel can represent. In our sample application, pixels in the images use 8 grayscale bits in the palette; thus, representing 2^8^ = 256 different colors. The goal of color quantization phase is to re-draw an image with a smaller number of colors that minimally impacts the quality of the image.

On the sensor network node’s perspective, by reducing the number of bits per pixel, the color quantization process can help reduce the size of the image. By reducing the number of per-pixel bits from eight to five (e.g., 32 colors), we can significantly reduce the image size, which in turn can also reduce the energy usage caused from image transmissions. Here, for simplification, we select the number of colors per palette to be a power of 2. As a result, by applying 3 - 7 bits per pixel (e.g., 8 - 128 colors), the absolute amount of data can be compressed by 38% to 88% of the original size. As we will show later, this reduction in image size leads directly to reduced radio on time on a resource-limited sensor node, thus increased node lifetimes.

**Algorithm 1** colorQuantization(image[w × h], bpp)

Color Quantization using fixed palette.

First **for** loop generates the palette

Second **for** loop replace each pixel to nearest color in the palette

Input: An Image, bits per pixel (bpp)

Output: Color quantized image

*palette[0]* = 2^7−*bpp*^ − 1

**for** n = 1 to 2^*bpp*^ − 1 **do**

 *palette[n] = palette[0]* + 2^8−bpp^*n

**for** pixel = 0 to w × h **do**

 *image[pixel] = get nearest centroid in palette*

**Return** *S*_*image*_

Note that there can be many ways of approaching a color palette for image drawing. The most popular approaches include fixed palette, median-cut palette, and palette using clustering algorithms such as Linde-Buzo-Gray (LBG) [25] or K-means clustering [26].

For example, the widely used median-cut color quantization starts from identifying the smallest box containing all the colors, and this box is repeatedly divided as the median point of the longest direction into smaller rectangle. This iteration is performed until *K* boxes remain and the averages of each boxes are used as the palette item. On the other hand, clustering based color quantization tries to identify centroids (a palette) among the original image’s color distribution. The goal of clustering is to partition the original color set (e.g., 256 in our case) into *K* centroids.

Generally, the goals of color quantization algorithms are mostly in minimizing the root mean square error (RMSE) and weighted root mean square error (WRMSE), which are the core performance metrics of color quantization [27]. Among many existing algorithms, the fixed palette algorithm is the least complex, but can result in a relatively high RMSE and WRMSE. Other approaches, in general, show relatively better performance, but require a higher level of computational overhead [27] given the complexity of computing the palette. For us, the higher computational costs naturally leads to higher energy consumption on the resource-limited sensor nodes. Thus, from our system’s perspective, it is most beneficial to use a fixed palette approach that is shared between the server and individual motes. This approach eliminates the complexity of sharing a newly computed palette between the nodes and the server.

The proposed color quantiation process, including the fixed palette construction, is detailed in Algorithm 1. First, to construct the fixed color palette which is represented as an arithmetic sequence, we set the first number of the palette arithmetic sequence as 2^(7 − *bpp*)^ − 1. This first element is selected so that an equal number of colors (e.g., among the 256 full color set) are uniformly associated with the *K* centroids we select. The first *for* loop in Algorithm 1 generates the remaining elements in the color palette’s arithmetic sequence. For example, for a target *K* of 8, the resulting color palette (or the arithmetic sequence) would be {15, 47, 79, 111, 143, 175, 207, 239}. As the second step, we take the color bits for each pixel and compute which centroid (among the *K*) is the nearest to this pixel value. Eq (1) presents the equation for this process. Once identifying the nearest centroid using Eq (2), we substitute the original color value to the value from the palette. Note that from Algorithm 1, the palette creation and sharing with the server is a one-time process introducing minimal amortized overhead.

$$\begin{array}{c} index=\underset{k}{\text{argmin}}\{|original\_image\left[i\right]\left[j\right]-palette\left[k\right]|\} \end{array}$$ (1)

$$\begin{array}{c} new\_image[i][j]=palette[index] \end{array}$$ (2)

**Fig 4:**
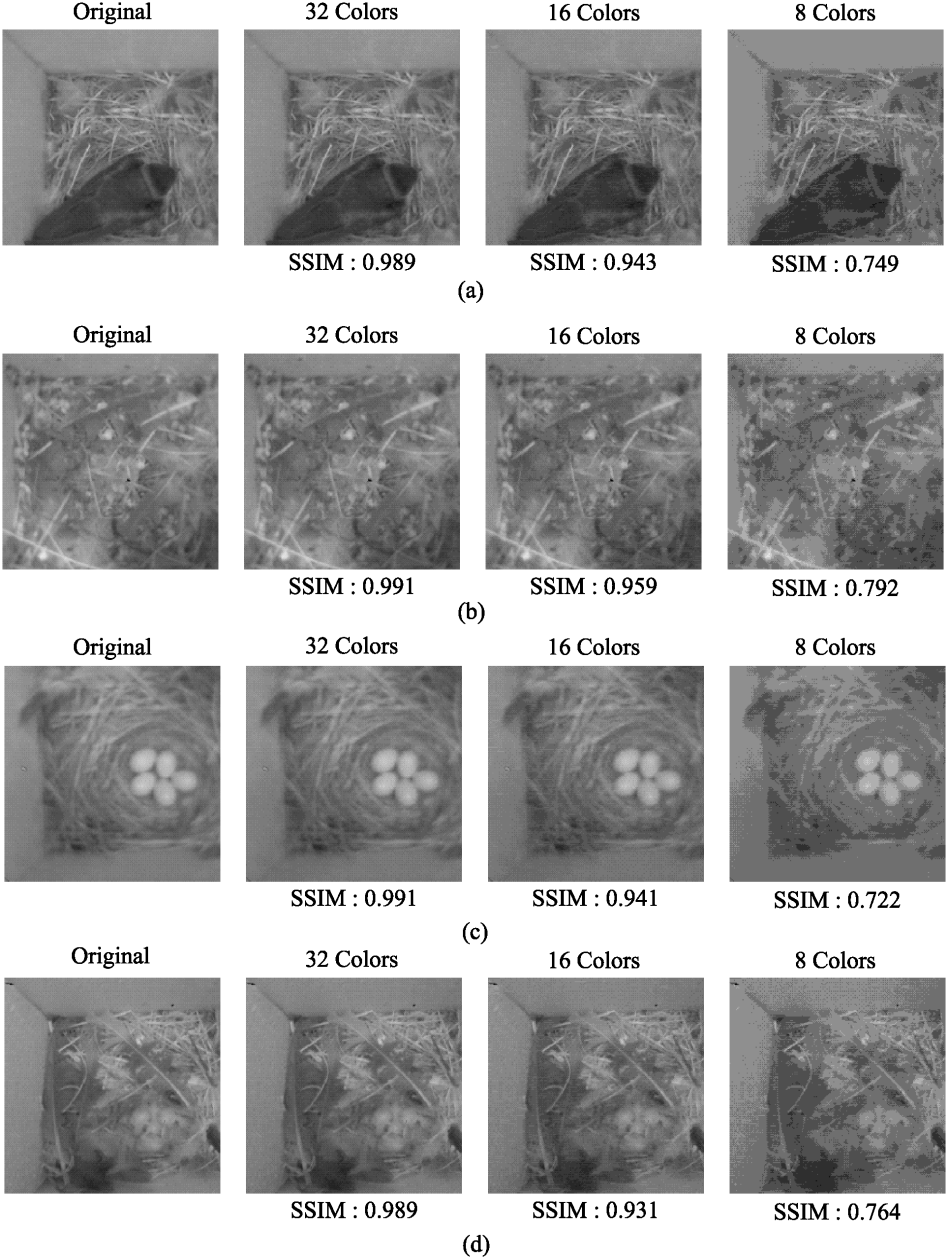
SSIM of sample images after color quantization with 32 colors, 16 colors, 8 colors on four classes of images: (a) Bird Presence (b) Empty nest (c) Egg Laying (d) Child Bird. The size images for each cases are, Original: 40KB, 32 colors: 25KB, 16 colors: 20KB and 8 colors: 15KB. Note: Original images are from [7].

To validate the effectiveness of our fixed palette approach, we compute the SSIM index to confirm how the color quantization process (negatively) impacts the quality of the image. Again, since the goal of our system is to run the images on a CNN, we focus on the SSIM index rather than the RMSE or WRMSE metrics. Fig 4 plots the SSIM index values after applying color quantization with a palette of three different color sets (32, 16, 8 colors) on sample images from the bird nest monitoring deployment. From the figures, we can see that 32, 16 color quantization has little impact on the image quality, maintaining a high SSIM of 0.95, where as using 8 colors would drop the SSIM to below 0.8.

### 3.2 Image resize

Adjusting the image size can be done in two different ways. One easier approach is to exploit the configuration capability of the camera module itself, if provided, to adaptively select between different image resolutions. However, this is effectively equal to sub-sampling the pixels without consideration for significance or magnitude of any given pixel. Furthermore, it excludes the possibility of obtaining the original full resolution image in case needed. The other approach is to take a full resolution image and post-process the image. Given that the images we collected from our target deployments are fixed in size, we also take a similar method of post-processing the pre-collected images to alter the image size.

**Fig 5:**
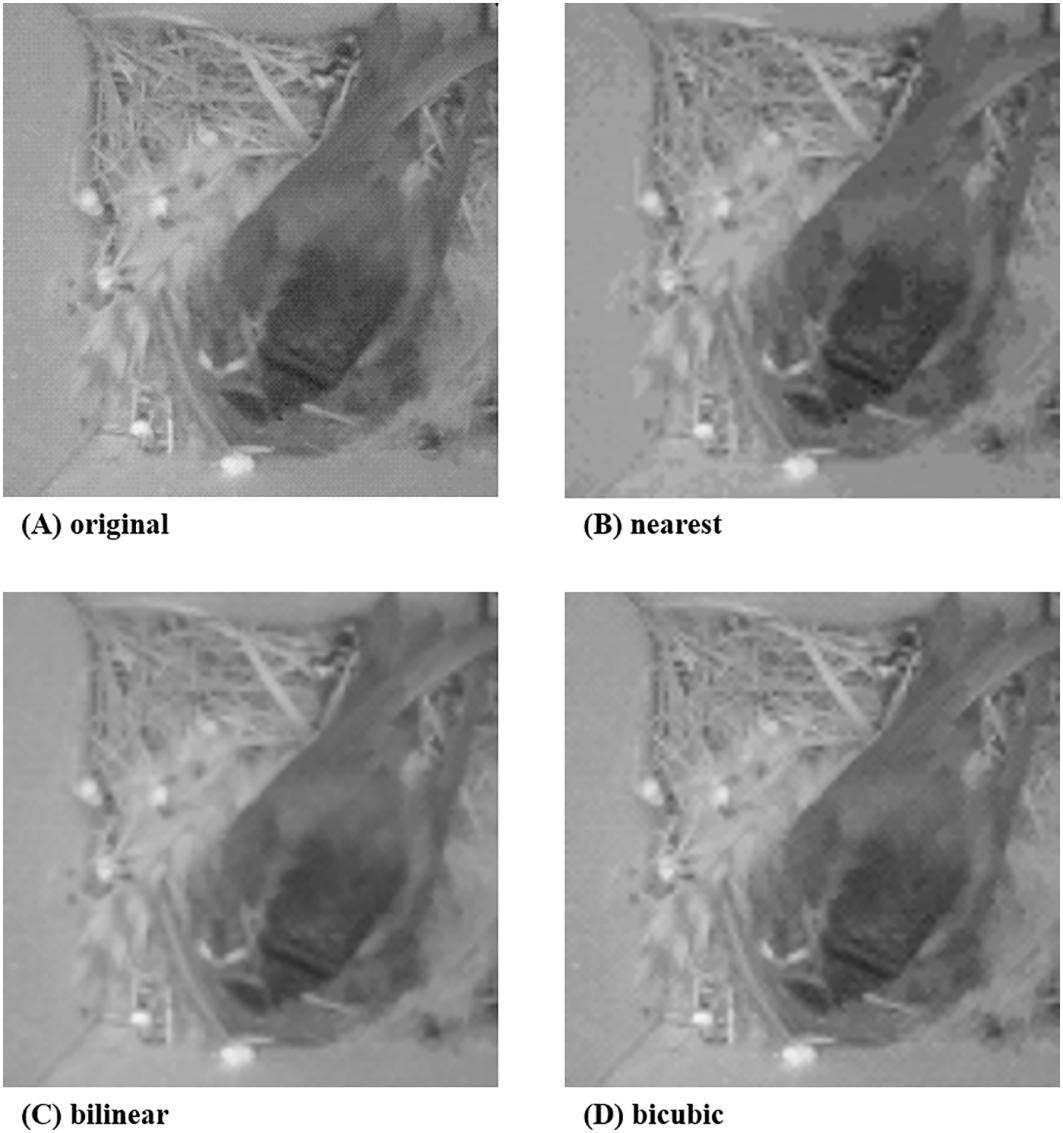
Results of image scaling by using (b) nearest neighbor, (c) bilinear and (d) bicubic interpolation, respectively, where (a) is the original sample image from [7].

Specifically, image resizing, or image scaling, is a geometric image transformation which modifies the image size based on an image interpolation algorithm. This image scaling process can increase or decrease the resolution of a target image so that the absolute size of image data is adjusted. The three most widely used algorithms for performing image interpolation are nearest-neighbor, bilinear, and bicubic interpolation [28]. The results from these three algorithms show differences in their characteristics such as quality, computational burden and memory requirements. Among these three algorithms, we select to use the bilinear interpolation algorithm for our image scaling process. As we show in Fig 5, bilinear interpolation-based image scaling typically offers a better image quality performance (e.g., edge blurring, discontinuities in edges and checkerboard effects) compared to the nearest-neighbor method, but can show worse performance compared to the bicubic approach. On the contrary, bilinear interpolation can run with a lower computational cost and memory requirement than bicubic interpolation-based image scaling. As a result, we select to use the bilinear interpolation-based image scaling method, given that this touches the middle ground of the image quality and computations complexity trade off.

**Fig 6:**
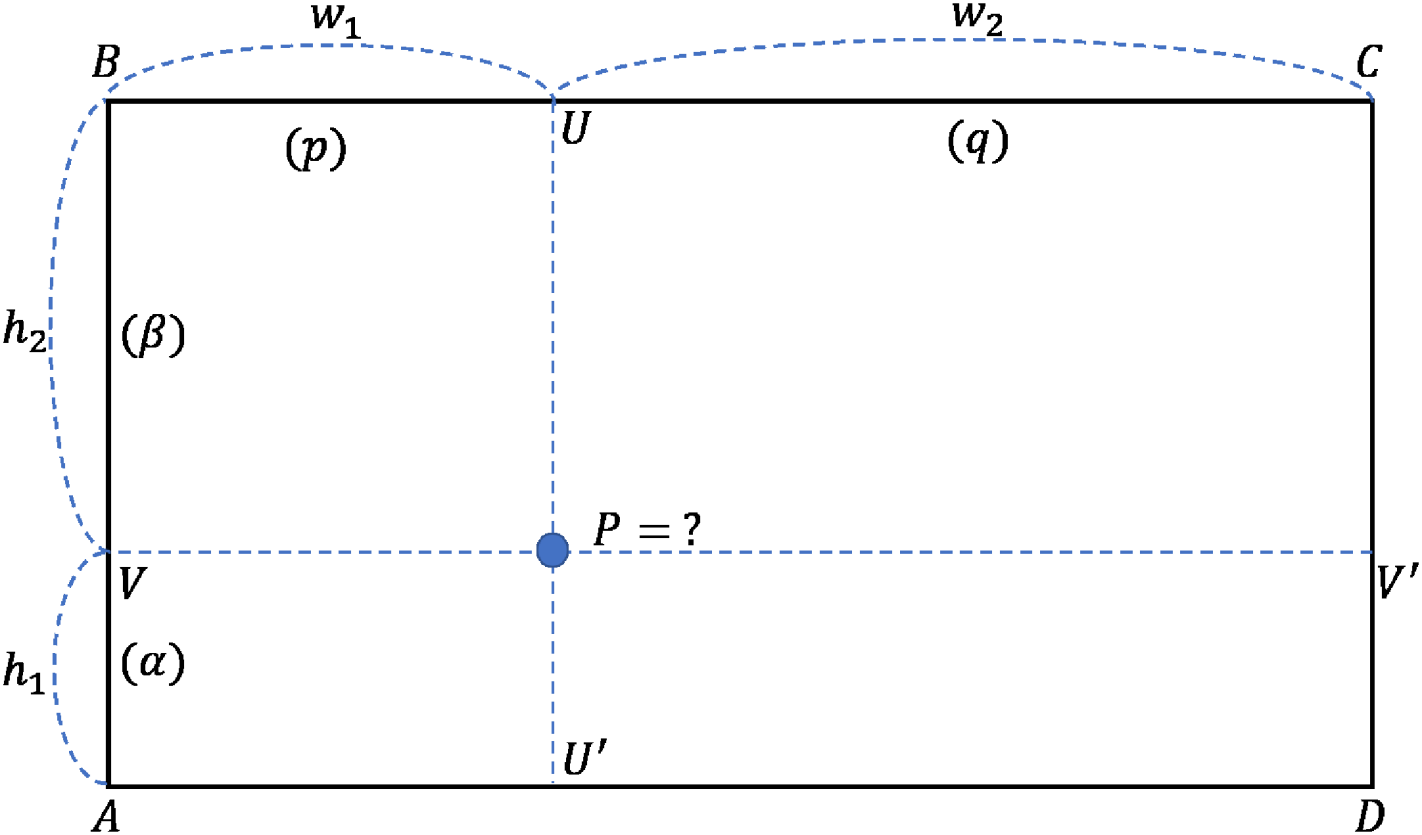
Illustration of a situation that calculates pixel P in □*ABCD* using bilinear interpolation.

When performing image scaling, bilinear interpolation computes a new pixel using a linear combination of four weighted input pixels. Fig 6 shows how bilinear interpolation works for interpolating a pixel *P* in □*ABCD*, which is composed of known pixels A, B, C and D. Here, *α* and *β* are the proportion of the height divided by *h*_1_ and *h*_2_ and similarly, *p* and *q* are the proportion of the width divided by *w*_1_ and *w*_2_.

$$\begin{array}{c} \alpha =\frac{h_{1}}{h_{1}+h_{2}},\;\;\beta =\frac{h_{2}}{h_{1}+h_{2}},\;\;p=\frac{w_{1}}{w_{1}+w_{2}},\;\;q=\frac{w_{2}}{w_{1}+w_{2}} \end{array}$$ (3)

Then, the pixel P can be calculated with Eq (4).

$$\begin{array}{ccc} V & = & q(\beta A+\alpha B),\\ V\prime & = & p(\beta D+\alpha C),\\ P & = & V+V\prime \end{array}$$ (4)

The principle of Eq (4) is that *V* is calculated using the interpolation between pixel *A* and pixel *B*, and *V′* is calculated with the interpolation between pixel *C* and pixel *D*. Finally, the interpolation between *V* and *V′*, pixel *P* is computed.

**Fig 7:**
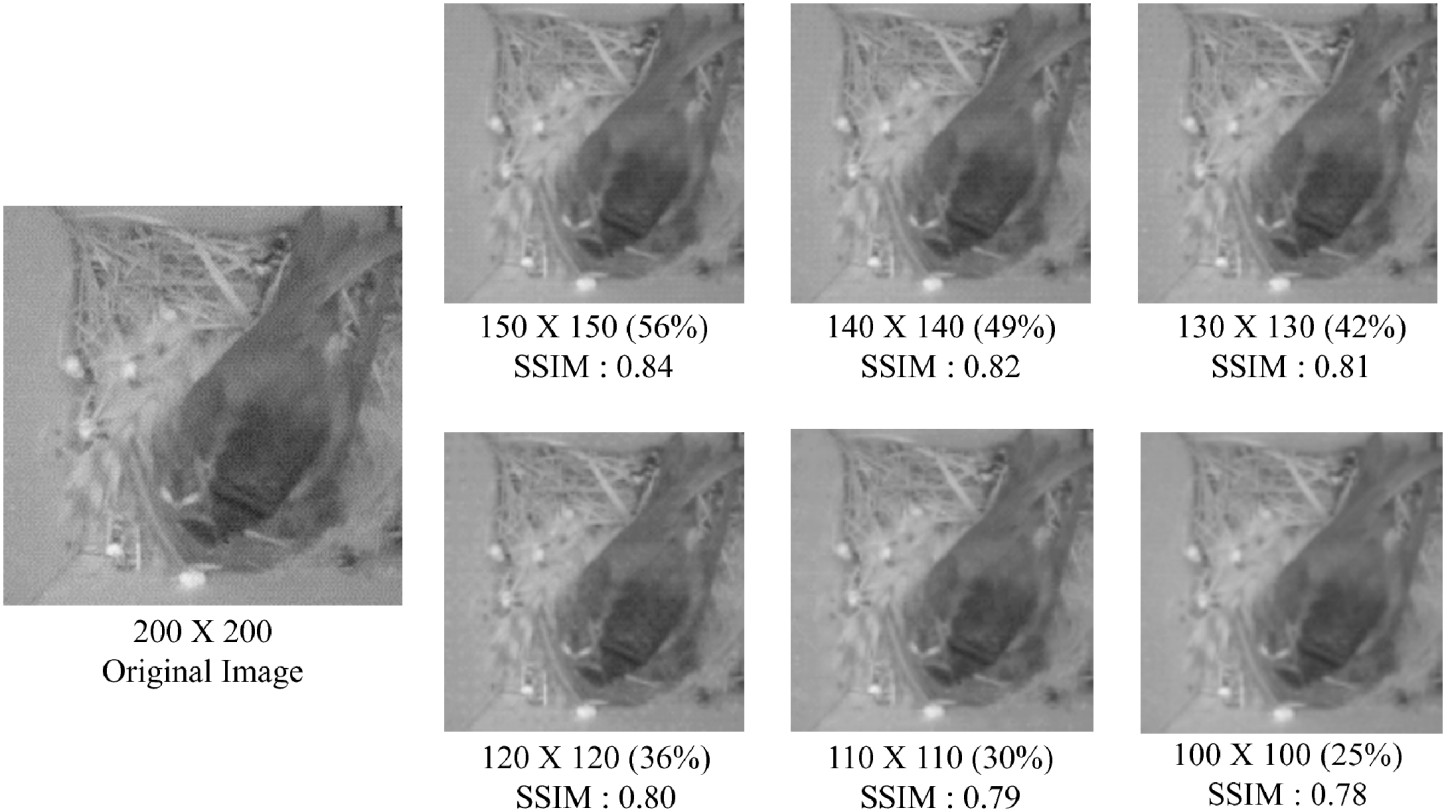
SSIM comparison between original 200 × 200 image and resized images. For calculating SSIM, we resize again back to original size 200 × 200. The percentage in parenthesis represents the amount of reduction in size. (Original image from [7].)

Fig 7 presents sample results from the image rescaling process. Here we show the SSIM index and the percentage of pixel counts compared to the original image size of 200 × 200. While the image scaling process maintains a high SSIM index of ∼0.8, the size of the image reduces significantly to as much as 25%.

**Fig 8:**
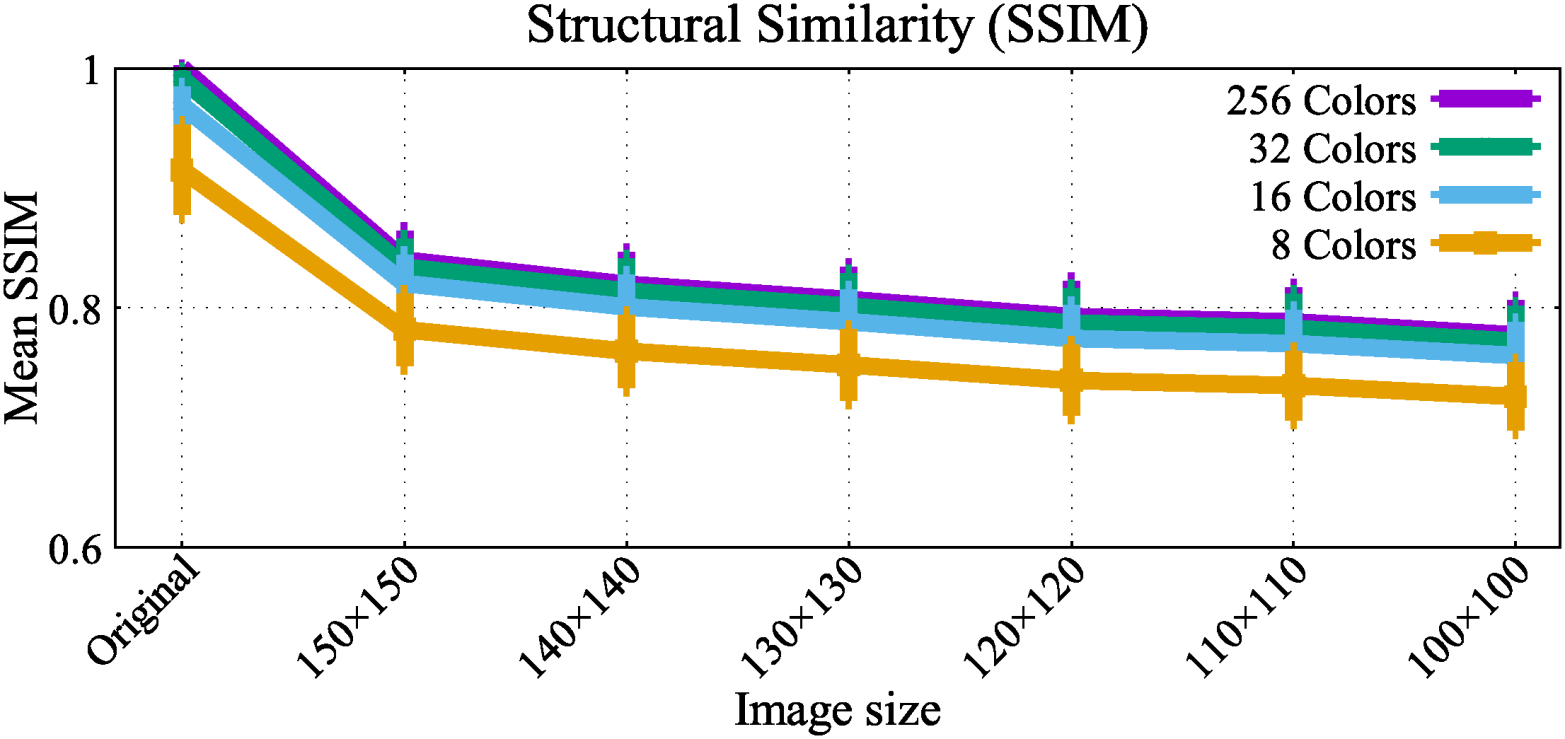
Mean SSIM between original images and modified images after color quantization.

Fig 8 presents the mean SSIM of the full image data set after performing both image resizing and color quantizaiton. Intuitively, when the image size reduces, the mean SSIM is also reduced monotonically. We can also observe here that 32 and 16 color quantization do not show significant quality differences compared to the 256 colored original image. The 8 color quantization, however, shows a more noticeable distortion compared to the other quantization levels.

Finally, another way to reduce the size of image data is to crop the image. Image cropping is, however, not appropriate for our system because of the following reasons. First, the cropped image should contain all of the meaningful features. To do this, the nodes need to self-identify the region of interest (ROI) within the image. Widely used algorithms to extract ROI can be classified into three types; thresholding, clustering and contour detection. Thresholding is the simplest method, and it is easy to implement with low computational cost. However, it requires a manually fine-tuned threshold parameter, which is difficult to configure in environment monitoring applications due to the sun ray changes over time, making the amount of illumination and distribution of light change continuously. Clustering and contour detection may show better results, but they require higher computational cost unsuitable for resource limited nodes. Second, cropping is not appropriate for CNNs. Training set for a CNN model has to contain the classes’ conventional shape. However, if cropping is performed incorrectly, the image may lose significant properties (shape, space occupied by feature in image) of themselves. For these reasons, we did not employ image cropping in our system design.

## 4 Computational cost and energy usage

Our findings until now show that using a combination of the color quantization and image scaling, we can effectively reduce the image sizes, and thus the amount of data that a wireless sensor node transmits. However, we note that the two image modification operations require a level of computational overhead to the resource limited embedded platform. In turn, this additional computational overhead induces energy usage as well. Therefore, it is important to confirm that the additional computational overhead does not negatively impact the lifetime of our devices. This section examines the impact of color quantization and image scaling on the computational cost and energy usage.

For our measurements, we use the Atmel SAM R21 XPlained board as the embedded sensor node platform [29], which is based on a system-on-chip combining an ARM Cortex-M0+ MCU and an IEEE 802.15.4 radio. Although it is not the identical platform used in the prior actual deployments, characteristics of the device are similar to those. Given that the SAM R21 board is used in a number of recent sensor network deployments [1], our measurements on this platform offers us a chance to observe how our findings can be employed in many newer sensor network deployments with advanced hardware specifications. To accurately measure the energy usage and the operation time of the various operations we are interested in, we use the Monsoon power monitor [30]. We then configure an experiment setting of quantizing image colors into a set of 8 to 256 colors, and resize the original 200 × 200 pixel images to as low as 100 × 100.

Table 1 shows the running time and energy usage on our SAM R21 embedded board for executing the color quantization and image resize processes. We can see here that the color quantization process does not introduce a significant amount of overhead, both in terms of operation time and energy usage. Specifically, the operational latency takes less than a millisecond itself. Note that our minimum unit of measurement is in milliseconds. As mentioned in Section 3.1, we use a fixed palette for color quantization; therefore, regardless of the quatization level, in the worst case of using a 200 × 200 pixel image, 40K integer comparison operations with the palette is the only computational operation required. This result suggests that the added overhead of using color quantization is negligible; thus, suitable for our system.

**Table 1:** Running time and energy usage measurements of color quantization and image resize operations from 200 × 200 to 100 × 100.

Operation		Operation Time (ms)	Energy Usage (mJ)
Color Quantization		<1	0.03
Image resize from 200 × 200	150 × 150	1670	66.11
	140 × 140	1463	57.96
	130 × 130	1262	50.01
	120 × 120	1064	42.15
	110 × 110	874	34.71
	100 × 100	755	29.96

On the other hand, Table 1 suggests that the image resizing operation shows a more variable performance in operation time and energy usage with respect to the target image size. The main cause of this latency is the time required to write the modified contents to the memory. Therefore, it is no surprise that the operation time and energy usage of the image scaling process is somewhat proportional to the target image size. We note that when performing color quantization by itself, the process would require a memory write phase as well. As a result, more time and energy would be needed to complete the process. However, when combining with the image scaling operations, the added latency and energy overhead will be minimal as the results in Table 1 imply.

**Fig 9:**
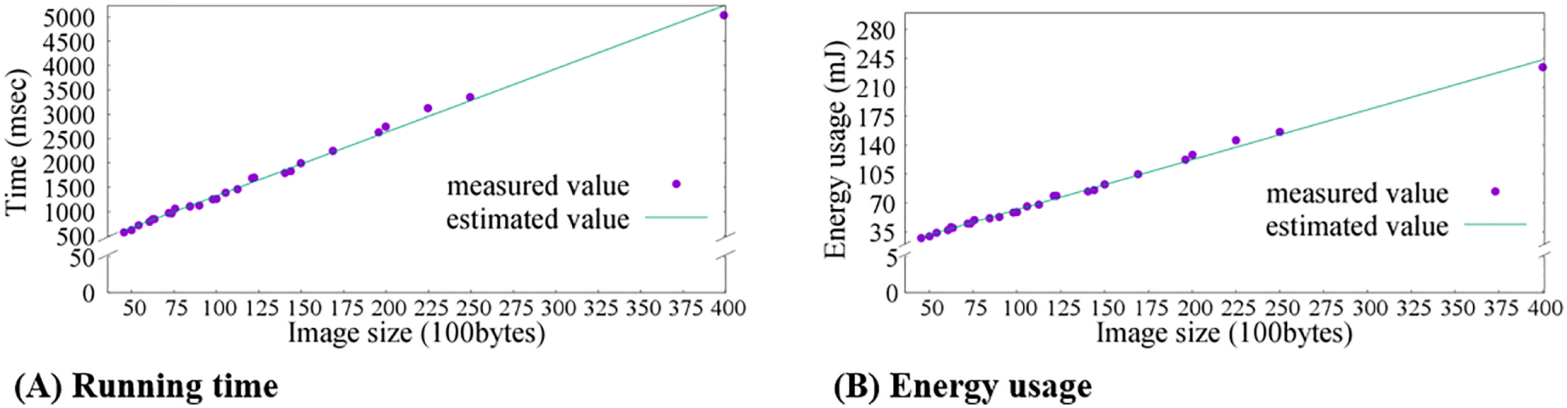
Image transmission latency and energy usage for differently compressed images.

Table 2 shows the amount of time and energy used when transmitting images of different color quantization levels and sizes on the embedded board’s IEEE 802.15.4 radio. Fig 9 plots this result with a linear regression line.

**Table 2:** Operation time and energy usage measurements on wireless transmission of images with images sizes ranging from 200 × 200 to 100 × 100.

Transmit image size (row & col)	8 colors		16 colors	
	Operation Time (ms)	Energy Usage (mJ)	Operation Time (ms)	Energy Usage (mJ)
200	1991.8	92.38	2748.8	127.91
150	1101.4	51.20	1457.8	67.93
140	963.9	44.88	1250.9	58.50
130	846.9	39.67	1109.4	51.52
120	718	33.91	969.8	44.74
110	569.8	27.17	787.2	36.66
100	477.4	22.42	617.6	29.27
Transmit image size (row & col)	32 colors		256 colors	
	Operation Time (ms)	Energy Usage (mJ)	Operation Time (ms)	Energy Usage (mJ)
200	3353.6	155.89	5034.6	234.53
150	1792.6	83.96	3127.2	145.52
140	1695.2	78.39	2624.2	122.16
130	1382.9	65.48	2245	104.42
120	1121.6	52.98	1828.4	85.51
110	1057.2	49.13	1681.4	78.39
100	841.9	40.43	1257.4	59.00

These results together that there is a linearly increasing trend in both operation time and energy costs with increasing images sizes, which is an expected behavior. Furthermore, in this case, the color levels (e.g., bits per pixel) also have a heavy impact on the message transmission overhead.

Overall, these experimental results suggest the need to perform image compression, given that the energy savings from sending a lighter-weight image outweighs the costs to perform on-board pre-processing on the images. Take for example an image compressed to 100 × 100 pixels with 8 colors. The additional power cost would be ∼29.99 mJ (i.e., energy for scaling (∼29.96 mJ) plus color quantization (0.03 mJ)), whereas the gain in energy from reduced transmissions is more than 212.11 mJ. While being the most extreme case, we use this example to show that image pre-processing at embedded sensor nodes can be heavily beneficial.

## 5 Image classification

Until now, we have described how images are modified at the individual sensor nodes and have shown the impact on the nodes’ resource usage. We now focus on the operations that occur on the server side, and make sure that our image size reduction does not impact the application’s classification accuracy. As we mentioned earlier, we build a classification model at the server using a convolutional neural network (CNN). Given that the field of CNNs for image classification is well-studied, we focus on exploiting four of the existing, widely used CNN models; AlexNet [31], VGG-16 [32], GoogLeNet(Inception-V1) [33], ResNet-50 [34]. Table 3 is a summary of the different CNN architectures we used to evaluate the classification performance using differently compressed images.

**Table 3:** Characteristic of famous convolutional neural networks architectures.

	AlexNet	VGG-16	GoogLeNet (Inception V1)	ResNet-50
Input Size	227 × 227	224 × 224	224 × 224	224 × 224
# of convolutional layers	5	16	21	49
# of weights	2.3 M	14.7 M	6 M	23.5 M
# of Mult-Adds*	666 M	15.3 G	1.43 G	3.86 G
# of fully connected layers	3	3	1	1
# of weights, Mult-Adds	58.6 M	124 M	1 M	2 M
Total Weights	61 M	138 M	7 M	25.5 M
Total Mult-Adds	724 M	15.5 G	1.43 G	3.9 G

* Mult-Adds means that “Multiply” and “Add” operations.

We note that the current trend in CNNs, as per the operations of AlexNet [31], is to increase the depth of the neural networks. Deeper networks can offer higher classification accuracy due to the increased non-linearity of the model. However, on the downside, the model becomes difficult to optimize and the chances of overfitting tends in increase with deep networks [23]. Using empirical evaluations, we also investigate the impact of network depth on the classification performance.

### 5.1 Convolutional neural networks for image classification

In deep neural network paradigm, convolutional neural networks can be considered the mainstream classifier for image data classification. Despite the efforts from the research community to perform visual target recognition and classification using traditional machine learning methods, CNNs rapidly dominated the visual recognition domain with its high accuracy classification results. As in many deep neural network designs, CNNs require minimal *a priori* knowledge on the training data compared to traditional machine learning algorithms. This is a major advantage of using CNNs compared to pre-existing learning methods.

A CNN consists of multiple hidden layers and an input and an output layer. Hidden layers in a CNN consist of convolutional layers, pooling layers, fully connected layers and normalization layers. The input (in our case) is the target image to be classified and the output is the context of the bird nest within the image. In addition, there is a cost function used to find the most fitted set of parameters and activation functions to determine the final output.

Our system chooses to use a CNN-based classification model at the backend server to accurately identify the context of bird nests. This relieves us from the concern that CNNs are computationally heavy for sensor devices. Instead, given that CNNs are strong in classifying images of various types, we saw this as an opportunity in applying low-quality images to the training and classification data sets via the image scaling and color quantization procedures within the WISN. Another motivation of using CNNs at the server as the classification model is that the CNN can be easily extended towards more detailed detection of objects in the image, beyond the high-level context [35, 36]. For example, from our dataset, we can train the model to detect more detailed observations such as the posture of birds in their nests, or egg count, which can be useful information for the domain scientists [15].

### 5.2 Filtering corrupted images

**Fig 10:**
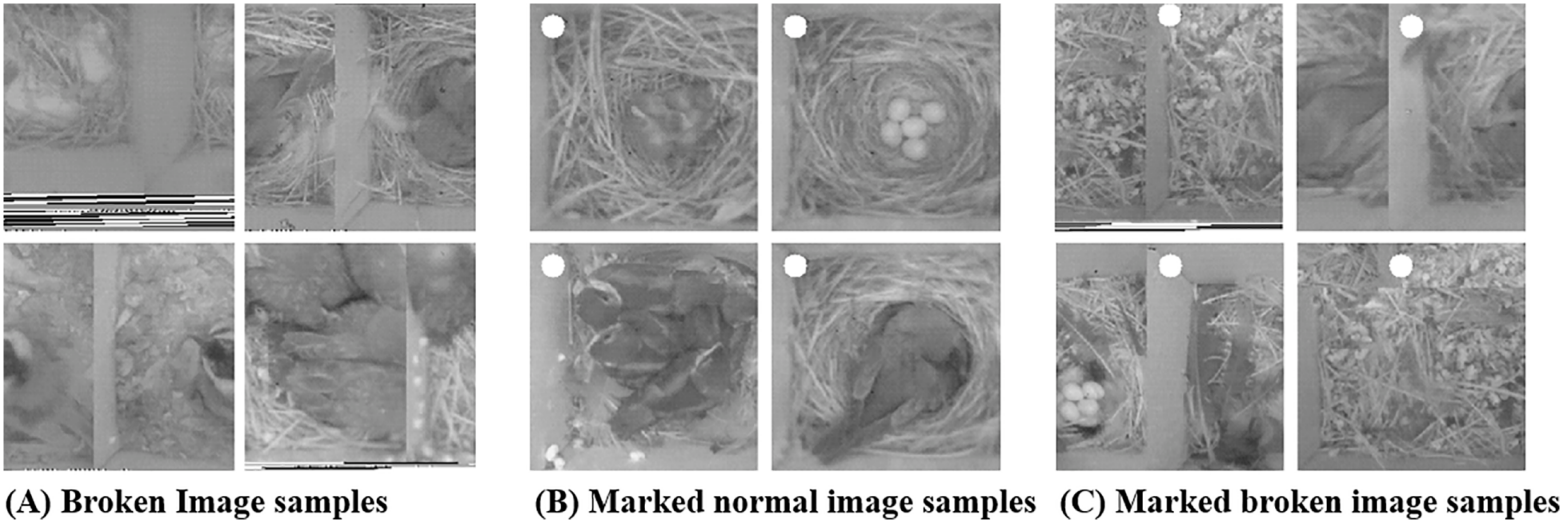
‘Broken’, ‘Marked Normal’, and ‘Marked Broken’ image samples from [7].

In wireless image sensor networks, due to limited resources at the individual sensor nodes, it is common to see a large number of “broken” images. For example, the limitations in the nodes’ RAM can result in images to overlap in corrupted patterns as they can be mis-aligned in the limited memory space. The training set data for the CNN will be mostly labeled by experts; thus, there is only a small chance of such broken images making it to the actual training data set. However, when in operation, the image context classification process should happen autonomously. As a result, there must be a way to identify broken images and filter these images from being classified. Fig 10A presents samples of images that are corrupted due to the mis-alignment of image pixels in the limited RAM.

In our system, filtering of these corrupted images occur in two steps. The first step occurs on the sensor nodes themselves. As a camera-generated image is temporarily stored at the sensor nodes’ RAM, nodes will draw a small white circle to the upper-right corner of the image. The location of where the circle should be plotted can be easily identified at the node given that the embedded camera’s image (pixel) size is already known. The diameter of this circle is $\frac{width}{10}$. For example, for the original 200 × 200 pixel images, the area of the circle is approximately ${\left(\frac{200}{10}\right)}^{2}*\pi \approx 1257\;pixels^{2}$, occupying approximately 3% of the entire image. Eventually, as the system operates, the images are sent to the server for context classification.

Before the CNN-based classification takes place, the second step of corrupted image filtering is performed. Specifically, the server, once receiving images from the sensor nodes, checks the location of this small white circle. As Fig 10B shows, if the images are not corrupted, the server will identify white circles at the upper-right corner of the images, whereas, for corrupted images as Fig 10C exemplifies, the location of the circle will be detected at a different location. This two phase process allows our system to easily identify corrupted images caused from memory alignment errors at the wireless image sensor nodes.

### 5.3 Building and evaluating the CNN model

Based on the classification criteria of previous work [15], we define four different contextual cases for the image set of our interest. Specifically, the target of our system is to classify the bird nest images into “Bird presence—Bluebird”, “Bird presence—Swallow”, “Empty nest”, “Egg Laying” and “Child Bird” states. Fig 4 shows sample images for each context state.

From the full data set, we select 60% of the images to be the training set, 20% as the validation set and 20% of the images as the test set. We note that we extracted a balanced number of images from different sensor nodes in the field. Since each node’s camera can have different background patterns (due to hardware installation differences or lighting effects), balanced selection of images allow the CNN model to well-understand the per-node image properties.

In terms of the quantity, images that are classified as “empty nest” or “bird presence” are dominant in the original 100K image data set. However, since images are sent to the server every ∼15 minutes and given the everyday living patterns of birds, there can be many images that are very similar in content. While the bird presence state images show some differences at the least, many of the “empty nest” images were almost identical and practically redundant. For this reason, we manually deleted 60K redundant empty nest images from the dataset. We summarize the actual quantity of data used in our work using Table 4. We note that having an asymmetric dataset can result in biased classification results. This is especially true if there is only a limited number of common patterns for images in a single class. Luckily, while our dataset is also asymmetic (c.f., Table 4), given that each class of images are uniquely distinct from the others (in most cases), we later show that our model does not face issues of biased classification results.

**Table 4:** Number image samples in the training, validation and test set for each class.

Class		Training Set (60%)	Validation Set (20%)	Test Set (20%)	Total (100%)
Bird	Swallow	6988	2329	2329	11646
	Bluebird	4166	1389	1388	6943
Child		2152	717	718	3587
Egg		1240	413	414	2067
Empty		9266	3089	3089	15444

For utilizing CNN classification on our server, we use the Caffe Deep Learning Framework [37]. In this environment, we train and test with four popular CNN models, AlexNet [31], VGG-16 [32], GoogLeNet [33] and ResNet-50 [34]. Our models are trained with four GPUs with 60 epochs for all network models and confirm that this is enough for all the models to converge. We also leverage transfer learning in our system design. The base idea of transfer learning is that we initially train a model using a base dataset and a base CNN architecture, then *transfer* the trained-weights for adapting to a target dataset. The most popular base dataset for images is ImageNet dataset [38], which contains 1.2 million images with 1000 categories. There are models pre-trained using popular CNN architecture with the ImageNet dataset in Model Zoo [39] for Caffe. Transfer learning is enticing to train a CNN model starting from a small dataset [40], train the model faster and give a chance to build a more accurate model. Specifically, the process of transfer learning can be summarized to two steps: (1) removing the final fully-connected layer of the base CNN architecture and (2) re-training the convolutional layer of pre-trained model in order to adapt the model to the target dataset and update the weights through a back-propagation process. In the training and validation of the model, we use four different data sets for each of the CNN models: one original image set and three color quantized (i.e. 32, 16 and 8 colors) image sets, all without image scaling (e.g., all image sizes are 200 × 200 pixels). Therefore, overall, we are left with 16 different CNN models to evaluate.

**Fig 11:**
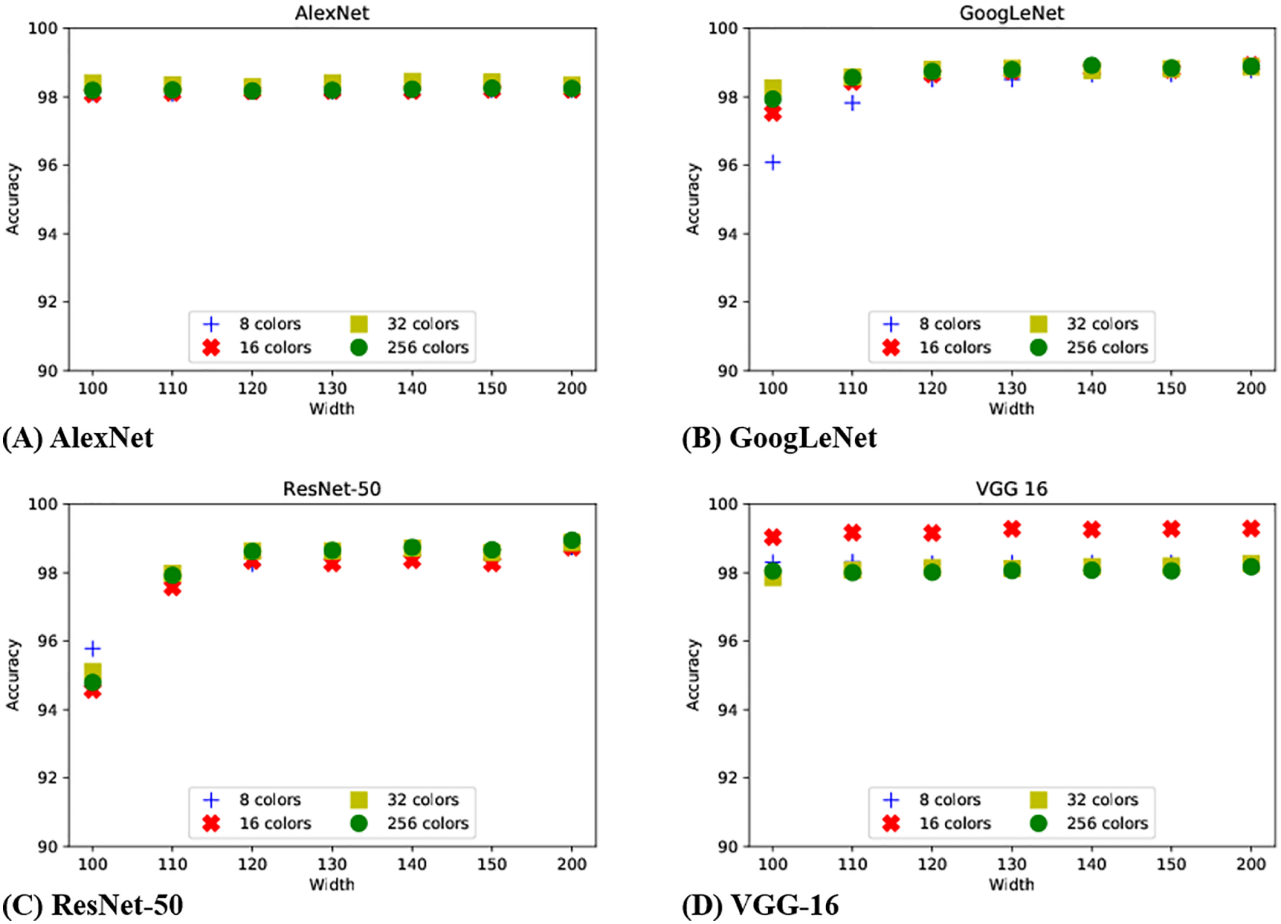
Classification results from four CNN models.

With the four different CNN models, we now generate 27 additional image data sets with 4 different bits per pixel (bpp) counts of 3, 4, 5 and 8 (8, 16, 32 and 256 colors), and 7 different width lengths 100, 110, 120, 130, 140, 150, and 200. The height of the image is set to be identical to the adjusted width. These data sets were generated using the test data, and were used to evaluate the performance of the four different CNN models we trained. Note that these values represent the average of three experimental runs. Fig 11 presents a summarized result on the accuracy of our four models with different bpp values. It can be seen that, regardless of the CNN model used, the accuracy results are high, being above 94% for all cases even for the most distorted image sets with 8 colors and 100 × 100 scaling. As a reference point, if we train and test the CNN model with original uncompressed data set, we achieved 98% classification accuracy.

In case of the two deepest network models, ResNet-50 and GoogLeNet, we can see from the results that when images are severely distorted (e.g., with heavy scaling or small bpp value), the classification accuracy decreases noticeably. The AlexNet-based CNN model, the most shallow network model (i.e., 5 layers), is less affected from the impact of image distortion. We conjecture that this is an effect caused from the number of fully-connected layers in the ResNet-50 and GoogLeNet models (e.g., 1 fully connected layer compared to 3 of AlexNet and VGG-16). Another interesting observation to make from this plot is that VGG-16 shows higher classification accuracy for images with 16 colors than any of the other cases. However, the difference in performance is small, and we plan to investigate deeper on why such accuracy was observed as part of our future work. Nevertheless, these results suggest that decreasing the image quality at the sensor nodes in order to reduce the transmission size and improve the energy efficiency of resource limited nodes, if well designed, does not severely impact the context classification performance at the server. We note that the results with the images scaled to size of 50 × 50 also showed similar trends with Fig 11 as well. While we focus on the object identification application for bird nests, other applications such as bird localization or segmentation within the nest may require higher-quality images. Nevertheless, minimal degradation of image quality (e.g., SSIM) suggests that our image compression approach can potentially be applied for other purposes as well. We leave the process of identifying image quality degradation limits while maintaining the accuracy as part of our future work.

**Fig 12:**
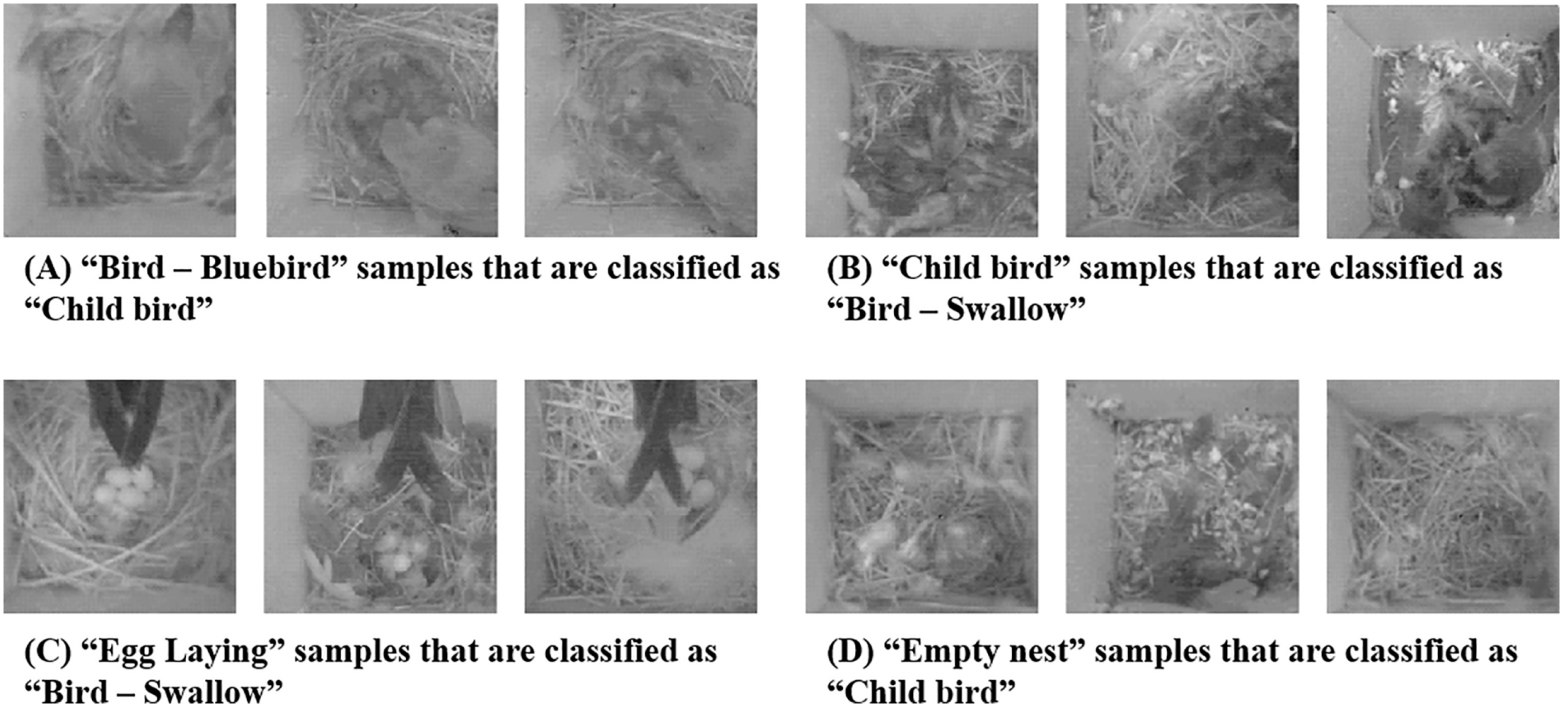
Sample mis-classification cases for 16 colors, 150 × 150 images. (a) the parent bird is feeding children birds. (b) children birds are growing to be adult birds. (c) there are eggs but a bird sits down on its nest and the tail causes mis-classification. (d) some “Empty Nest” images including feathers can be mis-classified as “Child bird”. Original images are from [7].

To gain additional intuition on the performance of our system design, we present in Table 5 the confusion matrix generated from the GoogLeNet model using test set processed as 16 color quantization and 150 × 150 image scaling. This test case considered a mid-level accuracy case, as we had cases with both higher and lower accuracy levels in our experiments. The first column in Table 5 show the ground truth labels along with the number of image samples included in each class. Note here that overall the classification was accurate, but similar looking images as we present in Fig 12 introduce some inaccuracies to our model.

**Table 5:** Confusion matrix of the classification results.

Ground Truth	Prediction				
Class	Bird - Swallow	Bird - Bluebird	Child Bird	Egg Laying	Empty Nest
Bird - Swallow (3717)	2294 (98.5%)	15 (0.6%)	14 (0.6%)	4 (0.2%)	2 (0.1%)
Bird - bluebird (3717)	4 (0.3%)	1381 (99.5%)	3 (0.2%)	0 (0.0%)	0 (0%)
Child Bird (718)	10 (1.4%)	20 (2.8%)	687 (95.7%)	1 (0.1%)	0 (0%)
Egg Laying (414)	4 (1.0%)	13 (3.1%)	5 (1.2%)	392 (94.7%)	0 (0%)
Empty Nest (3089)	0 (0.0%)	2 (0.1%)	0 (0.0%)	0 (0%)	3087 (99.9%)

Fig 12 presents representative mis-classified cases, with the classification result from the CNN and the ground truth context. Note from these images that most of the mis-classified cases were due to mixed classes of contexts (e.g., Fig 12A-12C). Furthermore, as we see in Fig 12D, objects within the image such as feathers and luminance issues are reasons behind mis-classification results that classify “Empty nest” images to “Child bird”.

## 6 Related work

Wireless image sensor networks have been used to monitor various environmental phenomena. As we have already detailed, the image dataset that we have used for this work is from the bird nest monitoring deployment in [8]. The work in [15] is another similar and earlier deployment for the bird nests at James Reserve, and it investigates use of machine learning techniques at the backend server for feature detection. There has been WISN for vineyard monitoring [41] and agricultural monitoring [42] as well.

In these wireless image sensor networks, image compression is one of the important issues for reducing energy consumption from wireless transmission. JPEG is widely used method of lossy image compression. However, it has been shown that the energy usage of image compression using JPEG outweighs the benefit of size reduction when ran on resource-limted platfroms [43]. JPEG 2000 [44] is another standard of image compression based on discrete wavelet transform (DWT) and embedded block coding with optimized truncation (EBCOT). JPEG 2000 shows better compression performance than JPEG by ∼20%, but typically at the cost of even higher computational overhead. Thus, there has been several work within WISN domain which attempted to design light-weight compression schemes suited for embedded platforms. Mostefa *et al.* [45] proposed an image compression technique using background subtraction. However, to determine that two images are different, the threshold had to be configured manually with domain knowledge. Furthermore, their proposal assumed indoor environment. In contrast, our target scenario is environmental monitoring where sun ray changes during a day. This would produce differences in the lightness of images periodically, and background subtraction would not work in this case. Paek *et al.* [46] proposed an image compression scheme using color quantization based on K-means clustering. K-means clustering based color quantization shows good compression ratio with reasonable image quality. However, the energy cost of K-means clustering outweighs the benefit from data reductions, and thus the work proposed to send raw images periodically, have the server compute the K centroids, and send the result back to the sensor nodes for compressing subsequent images.

Prior to the recent interest in deep neural networks, the majority of image classification works for wireless image sensor networks focused on feature extraction based machine learning with the help of domain experts. Recently with deep learning, the need for domain scientists’ hands-on efforts is decreasing. For example, the work by Ko et al. [15], classify the context of a bird nest using a SVM. Features in this work are detected using image processing techniques such as SIFT, and the classifier shows an accuracy of 82%. While this work is a pioneering work in bird nest monitoring wireless image sensor networks, our work argues that the improvement in data analytics models can help design a more efficient sensing system.

Recently, apart from sensor network domain, Mohanty *et al.* [47] proposed a CNN-based plant disease detection model. The authors annotate leaf images for 38 class labels and show results for how AlexNet and GoogLeNet perform with different pre-processing techniques. While similar to our work, this work does not consider images taken from embedded devices and do not take the resource limitations of wireless image sensor networks into account.

## 7 Conclusion

Autonomous image classification on top of wireless image sensor networks can enable various useful applications. However, with limited battery resources, running image classification algorithms on the sensor nodes or sending high-resolution raw images to the backend server are both burdensome tasks for embedded devices that has direct impact on the lifetime of the system. To address this problem, we propose an energy-efficient image processing mechanism combining image scaling and color quantization techniques that can significantly reduce the amount of data transferred on resource limited nodes, while maintaining the classification accuracy of the a CNN model at the backend server. Specifically, we have shown that, if well designed, an image in its highly compressed form can be well-classified with a CNN model trained in advance using adequately compressed training data. Our evaluation using a real image dataset shows that an embedded device can reduce the amount of transmitted data by ∼71% while maintaining a classification accuracy of ∼98%. When compared with the same conditions, this reduces the energy consumption by ∼71% compared to a WISN that sends the original raw images. We also identify that convolutional neural networks can operate robustly on top of lossy image compression algorithm, making them suitable for use in many sensor network deployments.
